# Urinary mRNA biomarker panel for the detection of urothelial carcinoma

**DOI:** 10.18632/oncotarget.9587

**Published:** 2016-05-25

**Authors:** Virginia Urquidi, Mandy Netherton, Evan Gomes-Giacoia, Daniel Serie, Jeanette Eckel-Passow, Charles J. Rosser, Steve Goodison

**Affiliations:** ^1^ Cancer Research Institute, MD Anderson Cancer Center, Orlando, FL, USA; ^2^ Department of Health Sciences Research, Mayo Clinic, Jacksonville, FL, USA; ^3^ Department of Health Sciences Research, Mayo Clinic, Rochester, MN, USA; ^4^ University of Hawaii Cancer Center, Honolulu, HI, USA; ^5^ Department of Urology, Mayo Clinic, Jacksonville, FL, USA

**Keywords:** diagnostic biomarkers, bladder cancer, multiplex, urinalysis, non-invasive

## Abstract

The early detection of bladder cancer is important as the disease has a high rate of recurrence and progression. The development of accurate, non-invasive urinary assays would greatly facilitate detection. In previous studies, we have reported the discovery and initial validation of mRNA biomarkers that may be applicable in this context. In this study, we evaluated the diagnostic performance of proposed molecular signatures in an independent cohort.

Forty-four mRNA transcripts were monitored blindly in urine samples obtained from a cohort of 196 subjects with known bladder disease status (89 with active BCa) using quantitative real-time PCR (RT-PCR). Statistical analyses defined associations of individual biomarkers with clinical data and the performance of predictive multivariate models was assessed using ROC curves. The majority of the candidate mRNA targets were confirmed as being associated with the presence of BCa over other clinical variables. Multivariate models identified an optimal 18-gene diagnostic signature that predicted the presence of BCa with a sensitivity of 85% and a specificity of 88% (AUC 0.935). Analysis of mRNA signatures in naturally micturated urine samples can provide valuable information for the evaluation of patients under investigation for BCa. Additional refinement and validation of promising multi-target signatures will support the development of accurate assays for the non-invasive detection and monitoring of BCa.

## INTRODUCTION

Being among the five most common malignancies worldwide, bladder cancer (BCa) is a major cause of morbidity and mortality [1, 2]. Although not typically life-threatening if detected early, more than 70% of patients with BCa will have a recurrence during the first two years after diagnosis. This recurrence phenomenon means patients face a lifetime of surveillance undergoing multiple invasive procedures. Current guidelines support a diagnostic approach of cystoscopy coupled with voided urine cytology (VUC). Invasive cystoscopy is associated with significant discomfort, possible infection and trauma. VUC is a non-invasive adjunct to cystoscopy, but the assay has poor sensitivity, especially for low-grade and low-stage tumors [1, 3]. Accordingly, a number of urine-based diagnostic assays have been developed commercially, but to date, these assays lack adequate accuracy to replace VUC or to support or guide cystoscopy. The development of accurate assays that can detect and monitor bladder cancer non-invasively through urinalysis would be a major advance, benefiting both patients and healthcare systems.

In previous studies [4, 5], we demonstrated the feasibility of profiling the transcriptome of urothelia obtained from naturally micturated urine and developed an analytical approach to identify cancer-associated gene signatures. Genome-wide expression and validation of selected candidate biomarkers in an independent cohort of subjects identified multiplex molecular signatures that achieved promising diagnostic performance. Here, we report the evaluation of a panel of candidate mRNA biomarkers compiled from our own studies and others [6–9], in a larger and more diverse independent cohort monitored using a quantitative real-time PCR (qPCR) platform.

A significant association with the presence of BCa was confirmed for the majority of our candidate diagnostic biomarkers [5], and for those reported by other groups [6–9]. Multivariate modeling identified an 18-target biomarker signature that achieved strong overall diagnostic performance (AUC 0.935), achieving 85% sensitivity and 88% specificity. This retrospective phase II biomarker development study [10] confirms the potential of using urothelial cell gene expression signatures for the non-invasive detection of BCa, and suggests that the described biomarkers and predictive models warrant further investigation with respect to developing an assay that could aid urology patient management.

## RESULTS

A panel of candidate mRNA biomarkers was monitored in an independent set of naturally voided urine samples. Twenty-one targets were selected from our previous discovery studies that identified a set of transcripts that were significantly associated with the presence of BCa [4, 5]. An additional 17 promising mRNA biomarkers from other BCa diagnostic studies [5–8] were also included, plus some targets identified through urinary proteomics [11, 12] or solid tumor tissue-based studies [13]. Urothelial cell samples were isolated from a total of 196 subjects, of which, 89 subjects had biopsy-proven BCa. Demographic and clinicopathological details of cases and controls are provided in Table 1. Gender distribution (3-4 times more men than women), the presence of gross hematuria (blood in the urine visible to the naked eye), and older patients in the BCa group reflect typical BCa incidence statistics [1].

**Table 1:** Demographic and clinicopathologic characteristics of study cohort

	Controls n=107		Cases n=89		*P*-value
**Median Age** (range, years)	59	(19-90)	70	(29-94)	0.0001
**Gender**					
Male	82	(7.6%)	75	(84.3%)	0.18
Female	25	(23.4%)	14	(15.7%)	
**Race**					
White	71	(66.3%)	70	(78.6%)	
African American	8	(7.4%)	7	(7.8%)	
Other	9	(8.4%)	8	(8.9%)	
Unknown	19		4		
**Cytology results**					
Missing	81		5		
Negative	19	(17.7%)	35	(49.3%)	
Reactive	4	(3.7%)	5	(7.0%)	
Suspicious	2	(1.8%)	3	(4.2%)	
Positive	1	(0.9%)	28	(39.4%)	
**Clinical stage**					
Tis	n/a		8	(9.5%)	
Ta	n/a		20	(23.8%)	
T1	n/a		18	(21.4%)	
T2	n/a		31	(36.9%)	
T3	n/a		7	(8.3%)	
**Grade**					
Missing	n/a		8		
Low	n/a		14	(17.3%)	
High	n/a		67	(82.7%)	
**Hematuria**					
Missing	8		2		
Yes	9	(9.1%)	27	(31.0%)	0.0002
No	90	(90.9%)	60	(69.0%)	

### Association of biomarkers with bladder cancer

RT-PCR analysis confirmed that the control gene transcripts were detected in all samples tested, however, as expected, candidate diagnostic markers were undetectable in a broad range of samples (Table 2). To avoid bias introduced by the issue of non-detects [14], we employed a left-censoring statistical approach to determine per-target differential expression in cases versus controls. Table 2 provides univariate differential expression results for each biomarker, ranked by Tobit model [15] *P*-value. The majority of the markers selected from urinary RNA-based, discovery and validation studies were confirmed as being significantly associated with the presence of BCa. Conversely, other than CTAG2, the biomarkers included from tissue-based studies [16], or those targets identified through urinary proteomics [13], were not significantly associated with disease. Additional information on biomarker candidacy was obtained by evaluating the association with specific clinical factors or distinct subsets of patients. Identified associations may impact decisions regarding inclusion in a test panel for a specific clinical utility. Left-censored Tobit models were used to estimate and compare associations of biomarkers with BCa and with clinical factors (hematuria, tumor grade, clinical stage, age, sex). Very few of the top-ranked candidate biomarkers (Tobit model *P* <0.05) were significantly associated with the presence of gross hematuria, or gender (Supplementary Table S2). While several biomarkers were weakly associated with age, a number of biomarkers did have significant associations with tumor grade and muscle-invasive disease (Supplementary Table S2).

**Table 2:** Univariate Tobit model results for testing the association of 44 candidate biomarkers with case-control status

Gene	Study [Ref]	% Samples Censored		Tobit Model	
		Controls n=107	Cases n=89	Estimate	*P*-value
SNAI2	Florida [5]	0.75	0.19	5.94	4.92E-13
IGF2	Barcelona [7]	0.29	0.02	4.75	2.07E-12
CA9	Florida	0.83	0.31	6.67	2.38E-10
MDK	Australasia [9]	0.19	0.03	3.25	1.45E-09
MMP12	Florida	0.23	0.07	3.33	5.70E-07
CRH	Barcelona	0.91	0.45	8.09	1.33E-06
KRT20	Barcelona	0.25	0.07	3.38	3.08E-06
PPP1R14D	Barcelona	0.72	0.25	3.62	3.42E-06
RAB1A	Florida	0.16	0.04	1.50	4.63E-06
TMEM45A	Florida	0.52	0.19	4.41	5.05E-06
MMP1	Florida	0.30	0.11	2.93	1.42E-05
SERPINE1	Florida	0.25	0.09	1.82	7.06E-05
MAGEA3	Barcelona	0.97	0.63**	11.01	7.96E-05
BIRC5	Florida	0.69	0.29	2.42	8.97E-05
MMP9	Florida	0.07	0.02	1.57	1.21E-04
POSTN	Barcelona	0.92	0.57**	4.91	2.74E-04
DMBT1	Florida	0.60	0.22	2.90	2.78E-04
DSC2	Florida	0.13	0.07	1.47	3.33E-04
ERBB2	Florida	0.07	0.02	1.44	6.21E-04
ANXA10	Barcelona	0.49	0.26	3.65	6.92E-04
SLC1A6	Barcelona	0.91	0.57**	4.25	8.01E-04
CCL18	Florida	0.50	0.17	2.48	1.19E-03
CTAG2	[13]	0.95	0.67**	9.88	1.58E-03
CDK1	Australasia	0.39	0.13	1.70	1.77E-03
HOXA13	Australasia	0.27	0.11	1.67	1.92E-03
CXCR2	Australasia	0.06	0.01	1.22	2.28E-03
CTSE	Barcelona	0.28	0.15	1.74	5.99E-03
SEMA3D	Florida	0.76	0.47	3.07	8.70E-03
KLF9	Barcelona	0.25	0.08	1.17	8.97E-03
VEGFA	Florida	0.00	0.01	0.49	1.17E-02
TERT	Barcelona	0.96	0.71**	3.46	1.74E-02
MMP10	Florida	0.24	0.15	1.49	3.66E-02
IGFBP5	Australasia	0.18	0.10	1.13	4.61E-02
CCNE2	Florida	0.32	0.12	0.75	6.10E-02
ANG	Florida	0.96	0.98**	−7.81	7.04E-02
SYNGR1	Florida	0.20	0.09	0.79	1.04E-01
CXCL1	[12]	0.02	0.01	0.49	1.42E-01
AHNAK2	Barcelona	0.37	0.15	−0.61	2.26E-01
IL8	[12]	0.00	0.00	0.54	2.32E-01
APOE	[12]	0.04	0.02	0.40	2.52E-01
AGT	Florida	0.61	0.37	−0.59	4.33E-01
PRAME	[13]	0.70	0.52**	0.81	5.56E-01
PLAU	[12]	0.03	0.03	−0.06	8.72E-01
MXRA8	Florida	0.66	0.35	0.00	9.94E-01

Biomarkers are ranked by Tobit model P-value. Because of censoring, the Tobit model estimate represents the difference between cases and controls in the un-observed latent variable. The percent of cases and controls censored is provided.

** Targets that were censored in >50% of cases.

### Multivariate analysis and prediction modeling

**Figure 1:**
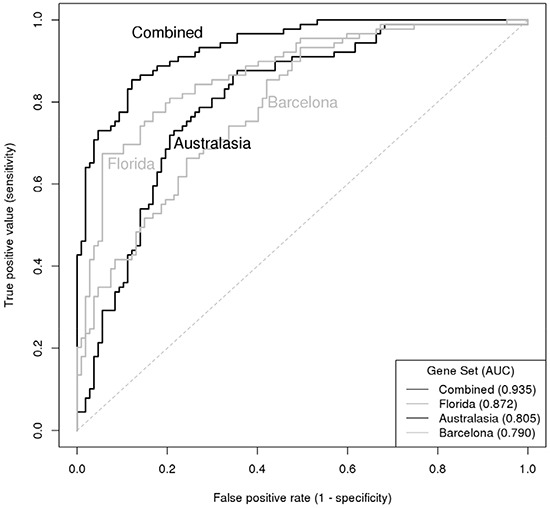
ROC curve illustrating the diagnostic accuracy of 4 gene set classifiers for predicting presence of bladder cancer Curves are presented for the Australasian panel [9], Barcelona panel [7], Florida panel [5] and the combination of all biomarkers.

To identify multifactorial gene sets that could predict the case-control status of a given sample, multivariate logistic models were constructed. Biomarkers that had a univariate Tobit model *P*-value <0.05, and were detectable in at least 50% of cases (Table 2) were included in the multivariate analyses. Predictive models were derived for three biomarker panels (*Australasia* [9], *Barcelona* [7]*, Florida* [5]), and for the combination of all markers. The LASSO approach [17] was used to shrink model coefficients, and model performance was described using receiver operating characteristic (ROC) analysis [18]. Corresponding odds ratios for the multivariate logistic regression models are shown in Table 3. An 18-gene prediction model derived from a combination (Table 3) of all biomarkers was optimal (Figure 1), achieving an AUC of 0.935 (optimism corrected AUC 0.878 [19]) with estimates of 85% sensitivity and 88% specificity [20]. Of the 71 cases that had VUC data available, VUC evaluation correctly detected 30 (49%) cases and the optimal predictive model identified 61 (86%) cases correctly.

**Table 3:** Multivariate logistic models using genes from 4 different panels

Gene	Multivariate Lasso Odds Ratios			
	Australasia	Barcelona	Florida	Combined
SNAI2			1.193	1.190
IGF2		1.168		1.291
CA9			1.110	1.165
MDK	1.247			1.312
MMP12			1.117	1.079
CRH		---		---
KRT20		1.048		1.072
PPP1R14D		---		---
RAB1A			1.306	1.090
TMEM45A			1.025	---
MMP1			1.049	1.113
SERPINE1			1.022	---
BIRC5			0.995	0.837
MMP9			1.128	1.211
DMBT1			---	---
DSC2			---	0.955
ERBB2			1.215	---
ANXA10		---		0.982
CCL18			0.959	0.990
CDK1	1.027			1.055
HOXA13	---			---
CXCR2	1.117		---	---
CTSE		---		0.991
SEMA3D			0.928	0.888
KLF9		1.050		1.130
VEGFA			---	---
MMP10			0.997	0.937
IGFBP5	---			---

Australasian panel [9], Barcelona panel [7], Florida panel [5], and the combination of all biomarkers (Combined). The Lasso method was used to shrink model coefficients; the corresponding odds ratios are provided.

## DISCUSSION

The development of accurate assays for the non-invasive detection of bladder cancer continues to be a challenge. A number of tests have been developed to detect tumor-associated urinary biomarkers, but due to poor sensitivity and overall accuracy, none of these assays have sufficient predictive power to be applied to the management of individual patients [21]. A shift from single biomarker assays [22] to multiplex molecular signatures that reflect the multiple pathways evident in BCa development provides an opportunity to develop assays with clinical utility for a breadth of diagnostic scenarios. In this study, we were able to confirm that a diagnostic panel of candidate mRNA biomarkers can accurately detect bladder cancer using a non-invasive urinary assay.

To look for BCa-associated mRNA signatures in bladder cancer, we previously applied a high-throughput molecular profiling and bioinformatics strategy to urothelial cell samples that are naturally shed from the bladder lining and can be readily recovered from urine [4]. The rationale for analyzing the shed urothelial component of urine was two-fold. Firstly, the analysis of the component that will be the analyte of a future assay is optimal. Secondly, the analyte enables comparison of samples collected from subjects with non-malignant conditions. Conversely, solid tissue samples are available from surgically excised material, but truly normal bladder tissues are rarely available. In a subsequent study, we extended the transcriptome profiling analyses to 92 patient samples and for an independent validation cohort converted the monitoring of specific mRNA transcripts on a customized quantitative PCR platform [5]. A European team also used the urothelial sample approach for derivation of a BCa-associated mRNA signature [7]. From a 384-gene test panel, Mengual *et al.* identified a 12-gene signature that achieved good accuracy in diagnosing BCa in an independent cohort utilizing quantitative RT-PCR. In a validation study the 12-gene signature achieved a sensitivity of 80% with 86% specificity for discrimination of patients with BCa from controls [8] and gene subsets were derived for the prediction of tumor aggressiveness. An Australasian group focused on the detection of free-mRNA in urine supernatant and identified a 4-gene signature that detected BCa at a specificity of up to 85% [8]. In subsequent studies, the mRNA urinary test achieved 62% sensitivity and outperformed two commercially available tests (NMP22 ELISA and BladderChek), and VUC [9], and an additional target (CXCR2) was included, reportedly to decrease false-positive rates [23]. Together these studies demonstrate that a multiplex quantitative PCR test on voided urine sample holds promise as a non-invasive urine-based assay in the clinical work-up of at risk patients for BCa. The candidate biomarker set tested in the current study was compiled from these previous investigations [5, 8, 23].

This study represents an additional step towards the derivation of an accurate RNA-based diagnostic test for BCa detection. The selected targets were quantitated in an independent cohort that, although also collected at a Florida institution, represents a patient population distinct from that used in our discovery studies [4, 5]. Furthermore, whereas we used healthy controls in our discovery phase studies, here the control cohort was composed of patients undergoing clinical work-up for potential BCa, including cystoscopy. Another difference between this and our previous investigations was the application of a more stringent statistical approach. Here, we flagged biomarkers that were not present in at least 50% of the cases. A biomarker that is not detectable in the majority of cases tested is unlikely to be a robust diagnostic factor in a clinical test. We also used a left-censoring approach [16] to handle RT-PCR non-detects; reactions that fail to produce a signal above an arbitrarily pre-specified minimum. These non-detects are typically treated as ‘missing’ data leading to biased inference, so it is beneficial to use approaches that can reduce such bias when validating candidate gene expression biomarkers. We subsequently used Tobit modeling to compare gene expression differences, because it is designed to estimate linear relationships between variables when there is censoring in the dependent variable [15].

Analyses confirmed that the majority (75%) of candidate biomarkers tested in the study cohort were strongly associated with the presence of BCa. Strong estimates and low *P*-values would be criteria for selecting the best biomarkers for further study, but the presence/absence rate in cases and control also provides valuable information. A target (*e.g.* SNAI2) that is absent in the majority of controls but present in the majority of cases would have good potential as a BCa detection biomarker. Conversely, a biomarker that is absent in both cases and controls may be less valuable (*e.g.* SEMA3D and CRH), even when differential expression was statistically significant. Another criterion for candidate biomarker selection can be the association with specific clinical variables. While association with age, gender or the presence of hematuria could negatively influence the inclusion of a biomarker for a broad spectrum BCa test, the inclusion of markers that are significantly associated with BCa *plus* stage or grade might provide additional information with regard to patient evaluation and management.

In both univariate and multivariate analyses, the Florida panel provided the majority of the top-ranked biomarkers. This is to be expected given the number of candidates in each original panel and the fact that the study cohort in this study was similar to that used in our discovery studies [4, 5]. The other biomarker panels were derived from cohorts composed from institutes in Spain or New Zealand and so would be expected to include biomarkers that are more associated with BCa in their specific populations, but several of the biomarkers tested translated to be of potential value in a US population. For overall BCa classification, the combined predictive model had a sensitivity of 85% and specificity of 88%. These values compare very favorably with the performance of cystoscopy and VUC, which both rely on high specificity for overall accuracy [24–27]. The performance also compare well with the existing urinary tests for BCa detection. To date, there are four urine tests that have received FDA approval for diagnostic clinical use (BTA-Stat, BTA-Trak, NMP22 POC device, UroVysion FISH test), and a couple of others with approval restricted to post-treatment monitoring [21]. In a meta-analysis of 57 studies [28], although specificity of the current diagnostic tests was in the range of 74% to 88%, none achieved a sensitivity >69%. The limiting factor for these tests may be the reliance on single biomarkers or the inclusion of chromosomal changes that are known to be restricted to a subset of BCa patients. There is clearly an ongoing need for more sensitive urinary tests for BCa detection.

We recognize that the study has several limitations. Although both cases and controls were collected consecutively, we only initiated molecular analyses when the balance of samples in each group approached 50%. Disease prevalence is typically considerably lower in urologic practice, so evaluation of the validation study cohort is likely to provide an overly optimistic assessment of the assay predictive value. While this study represents an advance over previous studies that used only healthy controls [4], as a tertiary care facility, we do tend to see more high-grade, high-stage disease, and as samples were collected prior to clinical evaluation for bladder cancer, other neoplastic urological conditions (prostate cancer, benign prostatic hyperplasia, kidney cancer) are under-represented in our study cohort. We also recognize that biomarker performance values derived from within discovery or limited validation studies can over-estimate their potential importance with respect to utility in an independent cohort. To address these issues, we are currently recruiting patients in an ongoing prospective study that will minimize selection bias, better represent urological disease prevalence, and evaluate potential confounding comorbidities. A large-scale prospective study will also facilitate the derivation of positive and negative predictive values, which physicians prefer to apply in clinical decision making. As we expand the cohorts to include the breadth of urology clinic visitors, we will also endeavor to derive predictive models that might work optimally for particular clinical scenarios, including primary diagnosis, follow-up surveillance or treatment response, and investigate to what extent a RNA-based diagnostic signature can provide actionable data alone, or in conjunction with current gold standard evaluations. The current study uses research-level methodology and reagents, so another important objective is to investigate the transfer of the RNA biomarkers to a robust platform that can be routinely performed in a clinical laboratory. There are issues with the measurement of RNA analytes. RNA is notoriously labile, and PCR amplification techniques have problems associated with molecular target structure and carry-over contamination, however, a number of RNA-based assays are being translated into tests that meet clinical laboratory standards [29]. Analytical improvements combined with the establishment of diagnostic thresholds and assay read-outs in diverse validation cohort studies are likely to increase the overall accuracy of sample analysis and patient evaluation.

With an estimated 77,000 new cases in 2016 [2], bladder cancer is a common neoplastic disease with a high rate of recurrence and progression. The recurrence phenomenon makes it one of the most prevalent cancers worldwide. The development of robust non-invasive, urine-based assay for the detection of BCa is clinically urgent. In this study, we have confirmed the association of a panel of biomarkers with the presence of BCa and identified diagnostic signatures that achieve encouraging values of sensitivity and specificity. The promising targets described in this study will be the focus of ongoing studies to achieve analytical optimization, and to investigate the potential added value of the multiplex assay if integrated into clinical decision making.

## MATERIALS AND METHODS

### Clinical sampling and processing

Under IRB approval and informed consent, urine samples and associated clinical information were consecutively collected from subjects visiting the urology clinic at MD Anderson Cancer Center at Orlando Health, Orlando, FL between 2011 and 2014. The study cohort consisted of 107 individuals with no evidence of active urothelial cell carcinoma (controls) and 89 individuals with newly diagnosed primary urothelial carcinoma (cases). All subjects underwent standard clinical work-up, including office cystoscopy, and the majority also had axial imaging of the abdomen and pelvis. For the bladder cancer case group, histological confirmation of urothelial carcinoma, including grade and stage was defined from excised tissue. A summary of clinical data is given in Table 1. Prior to any intrusive investigation or treatment, 30-50 ml of midstream voided urine was collected from each subject in a sterile cup and stored at 4°C until processing (<3 hrs.). Each sample was assigned a unique identifying number before laboratory processing. Urothelial cells were pelleted from the total urine sample by centrifugation (600 × *g*, 4°C, 5 min), rinsed in PBS, pelleted again, and frozen for storage at −80C. Total RNA was purified using Qiagen RNeasy kit with subsequent Qiagen DNase treatment. RNA samples were evaluated quantitatively and qualitatively using an Agilent Bioanalyzer 2000, before storage at −80°C as previously described [4, 5].

### Quantitative real-time PCR analysis

#### Custom array design

Taqman low density arrays (TLDAs) were constructed by Applied Biosystems (AB). The TLDA format is a 384-well system that uses standard TaqMan assays and enables automated loading and high-throughput analyses [16]. Targets included on the custom TLDA were *endogenous controls* - PPIA, GAPDH, UBC, PGK1, identified previously [5] using pooled urine samples on a TaqMan® Human Endogenous Control Array (Applied Biosystems PN 4367563), plus 21 mRNA biomarkers significantly associated with the presence of BCa in our previous studies [4], herein called the *Florida Panel* - BIRC5, ANG, CA9, AGT, DMBT1, ERBB2, CCNE2, SNAI2, MMP12, DSC2, TMEM45A, SYNGR1, MMP10, VEGFA, CCL18, SERPINE1, MMP1, MMP9, MXRA8, SEMA3D, and RAB1A. The gene expression assays for the targets described above were the same as those used in our previous studies [4, 5]. We also included 12 mRNA markers reported by Mengual *et al.* in studies that also used isolated urothelia samples for bladder cancer detection [7, 8], herein called the *Barcelona Panel* – TERT, KRT20, CRH, KFL9, MAGEA3, SLC1A6, POSTN, AHNAK2, ANXA10, CTSE, and PPP1R14D. The PCR primers for these targets were obtained from the associated articles [7, 8]. A 5-target mRNA urinary signature reported by a group from New Zealand [6, 9] was also included, herein called the *Australasia Panel* - CDK1, MDK, IGFBP5, HOXA13 and CXCR2. Finally, we included a few additional targets gleaned either from bladder tumor tissue studies – PRAME, CTAG2 [13] or from our urinary protein biomarker studies – APOE, IL8, PLAU and CXCL1 [11, 12]. PCR primer pairs for target amplification were obtained from published articles and selected from Applied Biosystems inventory. See Supplementary Table S1 for AB PCR assay ID details.

### cDNA synthesis and quantitative RT-PCR reactions

Complementary DNA was synthesized from 20 to 500 ng of total RNA, using the High Capacity cDNA Reverse Transcriptase Kit (Applied Biosystems, Foster City) following the manufacturer's instructions, with random primers in a total reaction volume of 20μl. A multiplex RT-PCR pre-amplification reaction was performed using the pooled 48 TaqMan Gene Expression Assays as described previously [5]. Assay reagents at 0.2X final concentration were combined with 7.5 μl of each cDNA sample and 15 μl of the TaqMan PreAmp Master Mix (2X) in a final volume of 30 μl. Thermal cycling conditions were as follows: initial hold at 95°C during 10 minutes; fourteen pre-amplification cycles of 15 seconds at 95°C and 4 minutes at 60°C and a final hold at 99.9°C for 10 minutes. Ten microliters of undiluted pre-amplification products was used in the subsequent singleplex qPCR amplification reactions, combined with 50 μl of 2× TaqMan Universal PCR MasterMix (AB) in a final volume of 100 μl, following manufacturer's instructions. One sample of Human Universal Reference Total cDNA (Clontech) was included as an inter-assay calibrator in each TLDA [30]. The reactions were run in a 7900HT Fast Real-Time PCR System (AB). RT-PCR amplification results were processed with RQ manager (AB). The baseline correction was manually checked for each target and the Ct threshold was set to 0.2 for every target across all plates. Samples used for downstream analysis were required to be positive for control genes. Targets deemed to be undetermined or absent (Ct >40) were given a Ct 40 value.

### Statistical analysis

Differences in clinical covariates between bladder cancer cases and non-malignant controls were evaluated via Chi-squared test and Wilcoxon Rank Sum test, as appropriate. For each gene, the percentage of samples that were censored (Ct value=40) was calculated for cases and controls separately. All four of the control genes had 100% observed data, that is, there were no Ct values <40. To determine the adequacy of each of these four genes as control genes, we used the t-statistic to compare cases and controls and observed that GAPDH and UBC were significantly associated with case-control status. Thus, only PGK1 and PPIA were used to normalize the 44 biomarkers of interest. To avoid biased inference caused by the issue of qPCR non-detects (Ct value=40), we used a left-censoring approach [14]. Ct values of 40 were substituted with the highest observed Ct value for a given gene. Ct values were then normalized by subtracting the average Ct value of the two endogenous control genes (PGK1 and PPIA) from each of the 44 genes of interest. For each gene, left-censored Tobit models [15] were used to test for differences in gene expression between cases and controls. As a sensitivity analysis, t-statistics were also performed in order to determine the influence of left-censoring on the analysis results (Supplementary Table S1). Multivariable logistic models were used to develop a signature to predict bladder cancer diagnosis. Genes where <50% of the cases were censored were considered in the multivariable models and lasso was used to shrink the model coefficients. ROC curves and associated AUCs were calculated to assess the performance of the multivariable models. The sensitivity and specificity associated with the maximum Youden index [20] was selected from each ROC curve. Left-censored Tobit models [15] were additionally used to evaluate associations between gene expression and clinical variables. Results with *P*<0.05 were deemed statistically significant.

## SUPPLEMENTARY TABLES






