# Supplementary material for: Urinary mRNA biomarker panel for the detection of urothelial carcinoma
Source: Oncotarget. 2016 May 25;7(25):38731–40. doi: 10.18632/oncotarget.9587 (PMC5122424; doi:10.18632/oncotarget.9587)
Supplement: Supplementary file 1 [file oncotarget-07-38731-s001.pdf]

Urinary mRNA biomarker panel for the detection of urothelial carcinoma

SUPPLEMENTARY TABLES

Supplementary Table S1: Univariate results for testing the association of each of the 44 candidate biomarkers with case-control status.

See Supplementary File 1

Supplementary Table S2: Association of biomarkers with clinical variables.

See Supplementary File 2
