# Supplementary material for: Urinary mRNA biomarker panel for the detection of urothelial carcinoma
Source: Oncotarget. 2016 May 25;7(25):38731–40. doi: 10.18632/oncotarget.9587 (PMC5122424; doi:10.18632/oncotarget.9587)
Supplement: Supplementary file 2 [file oncotarget-07-38731-s002.docx]

**Supplemental Table 1.** Univariate results for testing the association of each of the 44 candidate biomarkers with case-control status. Biomarkers were ranked by Tobit model *P*-value. The T-test results are additionally provided as a sensitivity analysis and represent results when a Ct value of 40 is treated as real data (*i.e.*, not as censored data). The t-test estimate denotes the difference between the average Ct value in the cases versus the average Ct value in the controls. The Tobit model estimate represents the difference between cases and controls in the un-observed latent variable. Thus, genes with minimal to no censoring will have Tobit model estimates similar to the t-test estimate, while the estimates for genes with extensive censoring will be increasingly different as the censoring rate increases. Biomarkers that were censored in >50% of cases, or had Tobit model estimate *P*-value >0.05, were not included in the multivariate analyses presented in Table 3.

| Gene | AB Assay ID | Number Censored | | Percent Censored | | Included in Multivariate Models | Left Censored  Tobit Model | | T-test | |
| --- | --- | --- | --- | --- | --- | --- | --- | --- | --- | --- |
|  |  | Controls n=107 | Cases n=89 | Controls | Cases |  | Estimate | *P*-value | Estimate | *P*-value |
| SNAI2 | Hs00161904_m1 | 80 | 17 | 0.75 | 0.19 | Yes | 5.94 | 4.92E-13 | 2.58 | 4.35E-08 |
| IGF2 | Hs00171254_m1 | 31 | 2 | 0.29 | 0.02 | Yes | 4.75 | 2.07E-12 | 4.02 | 2.69E-11 |
| CA9 | Hs00154208_m1 | 89 | 28 | 0.83 | 0.31 | Yes | 6.67 | 2.38E-10 | 1.74 | 6.46E-04 |
| MDK | Hs00171064_m1 | 20 | 3 | 0.19 | 0.03 | Yes | 3.25 | 1.45E-09 | 2.98 | 1.41E-09 |
| MMP12 | Hs00899662_m1 | 25 | 6 | 0.23 | 0.07 | Yes | 3.33 | 5.70E-07 | 2.91 | 5.88E-07 |
| CRH | Hs00174941_m1 | 97 | 40 | 0.91 | 0.45 | Yes | 8.09 | 1.33E-06 | 0.23 | 6.98E-01 |
| KRT20 | Hs00300643_m1 | 27 | 6 | 0.25 | 0.07 | Yes | 3.38 | 3.08E-06 | 2.84 | 4.79E-06 |
| PPP1R14D | Hs00214613_m1 | 77 | 22 | 0.72 | 0.25 | Yes | 3.62 | 3.42E-06 | 0.60 | 2.15E-01 |
| RAB1A | Hs00366313_m1 | 17 | 4 | 0.16 | 0.04 | Yes | 1.50 | 4.63E-06 | 1.33 | 1.83E-05 |
| TMEM45A | Hs01046616_m1 | 56 | 17 | 0.52 | 0.19 | Yes | 4.41 | 5.05E-06 | 2.60 | 4.57E-05 |
| MMP1 | Hs00233958_m1 | 32 | 10 | 0.30 | 0.11 | Yes | 2.93 | 1.42E-05 | 2.32 | 2.82E-05 |
| SERPINE1 | Hs01126606_m1 | 27 | 8 | 0.25 | 0.09 | Yes | 1.82 | 7.06E-05 | 1.53 | 8.25E-05 |
| MAGEA3 | Hs00366532_m1 | 104 | 56 | 0.97 | 0.63 | No, EC | 11.01 | 7.96E-05 | -1.04 | 8.02E-02 |
| BIRC5 | Hs03043576_m1 | 74 | 26 | 0.69 | 0.29 | Yes | 2.42 | 8.97E-05 | 0.31 | 4.22E-01 |
| MMP9 | Hs00234579_m1 | 8 | 2 | 0.07 | 0.02 | Yes | 1.57 | 1.21E-04 | 1.51 | 1.47E-04 |
| POSTN | Hs00170815_m1 | 98 | 51 | 0.92 | 0.57 | No, EC | 4.91 | 2.74E-04 | -1.05 | 3.58E-02 |
| DMBT1 | Hs00244838_m1 | 64 | 20 | 0.60 | 0.22 | Yes | 2.90 | 2.78E-04 | 0.96 | 5.46E-02 |
| DSC2 | Hs00245200_m1 | 14 | 6 | 0.13 | 0.07 | Yes | 1.47 | 3.33E-04 | 1.37 | 2.31E-04 |
| ERBB2 | Hs01001582_m1 | 7 | 2 | 0.07 | 0.02 | Yes | 1.44 | 6.21E-04 | 1.40 | 4.84E-04 |
| ANXA10 | Hs00200464_m1 | 52 | 23 | 0.49 | 0.26 | Yes | 3.65 | 6.92E-04 | 2.09 | 3.35E-03 |
| SLC1A6 | Hs00192604_m1 | 97 | 51 | 0.91 | 0.57 | No, EC | 4.25 | 8.01E-04 | -1.47 | 4.66E-03 |
| CCL18 | Hs00268113_m1 | 54 | 15 | 0.50 | 0.17 | Yes | 2.48 | 1.19E-03 | 1.05 | 4.17E-02 |
| CTAG2 | Hs00535628_m1 | 102 | 60 | 0.95 | 0.67 | No, EC | 9.88 | 1.58E-03 | -1.31 | 4.39E-02 |
| CDK1 | Hs00938777_m1 | 42 | 12 | 0.39 | 0.13 | Yes | 1.70 | 1.77E-03 | 1.00 | 1.35E-02 |
| HOXA13 | Hs00426284_m1 | 29 | 10 | 0.27 | 0.11 | Yes | 1.67 | 1.92E-03 | 1.29 | 3.49E-03 |
| CXCR2 | Hs00174304_m1 | 6 | 1 | 0.06 | 0.01 | Yes | 1.22 | 2.28E-03 | 1.17 | 3.69E-03 |
| CTSE | Hs00157213_m1 | 30 | 13 | 0.28 | 0.15 | Yes | 1.74 | 5.99E-03 | 1.29 | 1.22E-02 |
| SEMA3D | Hs00380877_m1 | 81 | 42 | 0.76 | 0.47 | Yes | 3.07 | 8.70E-03 | -0.24 | 6.55E-01 |
| KLF9 | Hs00230918_m1 | 27 | 7 | 0.25 | 0.08 | Yes | 1.17 | 8.97E-03 | 0.89 | 1.43E-02 |
| VEGFA | Hs00900055_m1 | 0 | 1 | 0.00 | 0.01 | Yes | 0.49 | 1.17E-02 | 0.49 | 1.34E-02 |
| TERT | Hs00162669_m1 | 103 | 63 | 0.96 | 0.71 | No, EC | 3.46 | 1.74E-02 | -2.65 | 4.47E-07 |
| MMP10 | Hs00233987_m1 | 26 | 13 | 0.24 | 0.15 | Yes | 1.49 | 3.66E-02 | 1.14 | 5.16E-02 |
| IGFBP5 | Hs00181213_m1 | 19 | 9 | 0.18 | 0.10 | Yes | 1.13 | 4.61E-02 | 0.95 | 5.65E-02 |
| CCNE2 | Hs00372959_m1 | 34 | 11 | 0.32 | 0.12 | No, TLS | 0.75 | 6.10E-02 | 0.42 | 1.78E-01 |
| ANG | Hs01590076_m1 | 103 | 87 | 0.96 | 0.98 | No, TLS | -7.81 | 7.04E-02 | -3.91 | 5.82E-11 |
| SYNGR1 | Hs00377475_m1 | 21 | 8 | 0.20 | 0.09 | No, TLS | 0.79 | 1.04E-01 | 0.62 | 1.35E-01 |
| CXCL1 | Hs00605382_gH | 2 | 1 | 0.02 | 0.01 | No, TLS | 0.49 | 1.42E-01 | 0.49 | 1.48E-01 |
| AHNAK2 | Hs00292832_m1 | 40 | 13 | 0.37 | 0.15 | No, TLS | -0.61 | 2.26E-01 | -1.27 | 1.98E-03 |
| IL8 | Hs00174103_m1 | 0 | 0 | 0.00 | 0.00 | No, TLS | 0.54 | 2.32E-01 | 0.54 | 2.46E-01 |
| APOE | Hs00171168_m1 | 4 | 2 | 0.04 | 0.02 | No, TLS | 0.40 | 2.52E-01 | 0.39 | 2.49E-01 |
| AGT | Hs00174854_m1 | 65 | 33 | 0.61 | 0.37 | No, TLS | -0.59 | 4.33E-01 | -1.82 | 1.14E-04 |
| PRAME | Hs01022301_m1 | 75 | 46 | 0.70 | 0.52 | No, TLS | 0.81 | 5.56E-01 | -1.52 | 1.78E-02 |
| PLAU | Hs00170182_m1 | 3 | 3 | 0.03 | 0.03 | No, TLS | -0.06 | 8.72E-01 | -0.06 | 8.74E-01 |
| MXRA8 | Hs00260584_m1 | 71 | 31 | 0.66 | 0.35 | No, TLS | 0.00 | 9.94E-01 | -1.44 | 1.52E-04 |

AB: Applied Biosystems; EC: Excessive Censoring; TLS: Tobit Lacks Significance.
