# Supplementary material for: Urinary mRNA biomarker panel for the detection of urothelial carcinoma
Source: Oncotarget. 2016 May 25;7(25):38731–40. doi: 10.18632/oncotarget.9587 (PMC5122424; doi:10.18632/oncotarget.9587)
Supplement: Supplementary file 3 [file oncotarget-07-38731-s003.docx]

**Supplemental Table 2.** Association of biomarkers with clinical variables. Biomarkers are ranked by the *P*-value of the Tobit model estimate (difference in mean expression between cases and controls) of association with bladder cancer. Hematuria; association with the presence of gross hematuria. Grade; association with high-grade versus low grade. Muscle-invasive disease; stage information was grouped (non-invasive stages Tis, Ta, T1 versus invasive stages T2, T3) to indicate muscle-invasive disease.

|  | Association with Bladder Cancer | | Hematuria | | Tumor Grade | | Muscle Invasive Disease | | Age | | Sex | |
| --- | --- | --- | --- | --- | --- | --- | --- | --- | --- | --- | --- | --- |
| Gene | Estimate | *P*-value | Estimate | *P*-value | Estimate | *P*-value | Estimate | *P*-value | Estimate | *P*-value | Estimate | *P*-value |
| SNAI2 | 5.94 | 4.92E-13 | 0.883 | 2.96E-01 | 2.041 | 5.62E-02 | 2.351 | 1.10E-03 | 0.032 | 3.82E-01 | 1.320 | 2.20E-01 |
| IGF2 | 4.75 | 2.07E-12 | 0.930 | 2.92E-01 | 0.178 | 8.78E-01 | -0.571 | 5.05E-01 | 0.069 | 7.02E-02 | 0.440 | 6.95E-01 |
| CA9 | 6.67 | 2.38E-10 | 2.566 | 2.73E-02 | 1.228 | 4.70E-01 | 0.178 | 8.77E-01 | 0.081 | 1.20E-01 | 1.955 | 2.08E-01 |
| MDK | 3.25 | 1.45E-09 | -0.352 | 6.06E-01 | -0.503 | 5.63E-01 | -0.417 | 5.17E-01 | 0.014 | 6.31E-01 | 0.044 | 9.59E-01 |
| MMP12 | 3.33 | 5.70E-07 | -1.889 | 2.71E-02 | 1.891 | 1.02E-01 | 1.338 | 1.16E-01 | 0.019 | 6.21E-01 | -0.992 | 3.71E-01 |
| CRH | 8.09 | 1.33E-06 | 1.748 | 2.93E-01 | 2.388 | 2.94E-01 | 1.297 | 3.96E-01 | 0.251 | 6.08E-04 | 0.244 | 9.08E-01 |
| KRT20 | 3.38 | 3.08E-06 | 1.526 | 9.65E-02 | 2.091 | 7.55E-02 | 0.315 | 7.21E-01 | 0.074 | 5.91E-02 | -0.209 | 8.57E-01 |
| PPP1R14D | 3.62 | 3.42E-06 | 2.015 | 6.13E-02 | 2.107 | 1.85E-01 | 0.481 | 6.54E-01 | 0.121 | 8.36E-03 | 1.018 | 4.61E-01 |
| RAB1A | 1.50 | 4.63E-06 | -0.655 | 2.07E-01 | 0.602 | 3.93E-01 | -0.297 | 5.65E-01 | 0.013 | 5.71E-01 | 0.645 | 3.32E-01 |
| TMEM45A | 4.41 | 5.05E-06 | -0.143 | 9.07E-01 | 1.631 | 2.96E-01 | 1.973 | 7.48E-02 | 0.072 | 1.58E-01 | 0.810 | 5.93E-01 |
| MMP1 | 2.93 | 1.42E-05 | -0.693 | 4.36E-01 | 2.428 | 2.84E-02 | 2.291 | 3.50E-03 | 0.017 | 6.67E-01 | -0.753 | 5.11E-01 |
| SERPINE1 | 1.82 | 7.06E-05 | -0.163 | 7.91E-01 | 0.995 | 2.05E-01 | 0.254 | 6.57E-01 | 0.070 | 6.47E-03 | 0.212 | 7.84E-01 |
| MAGEA3 | 11.01 | 7.96E-05 | -1.031 | 6.44E-01 | 8.685 | 3.66E-02 | 2.503 | 2.34E-01 | 0.312 | 5.26E-03 | -1.813 | 5.17E-01 |
| BIRC5 | 2.42 | 8.97E-05 | 1.489 | 5.46E-02 | 1.765 | 1.29E-01 | 0.790 | 3.02E-01 | 0.069 | 3.68E-02 | -0.373 | 7.07E-01 |
| MMP9 | 1.57 | 1.21E-04 | -1.787 | 1.54E-03 | 2.293 | 3.19E-03 | 0.308 | 6.10E-01 | 0.001 | 9.60E-01 | -0.445 | 5.69E-01 |
| POSTN | 4.91 | 2.74E-04 | 2.684 | 4.54E-02 | 6.798 | 1.26E-02 | 3.406 | 8.08E-03 | -0.034 | 5.84E-01 | 0.989 | 6.04E-01 |
| DMBT1 | 2.90 | 2.78E-04 | -0.558 | 5.56E-01 | 1.231 | 3.41E-01 | 0.040 | 9.65E-01 | 0.073 | 7.04E-02 | 1.388 | 2.49E-01 |
| DSC2 | 1.47 | 3.33E-04 | -0.650 | 2.55E-01 | 4.244 | 2.44E-12 | 0.992 | 6.66E-02 | -0.008 | 7.37E-01 | 0.169 | 8.15E-01 |
| ERBB2 | 1.44 | 6.21E-04 | 0.971 | 7.93E-02 | 0.957 | 1.81E-01 | 0.090 | 8.67E-01 | 0.043 | 6.76E-02 | 1.051 | 1.31E-01 |
| ANXA10 | 3.65 | 6.92E-04 | 3.300 | 3.18E-02 | 2.722 | 2.07E-01 | -0.192 | 8.98E-01 | 0.077 | 2.46E-01 | 2.326 | 2.44E-01 |
| SLC1A6 | 4.25 | 8.01E-04 | 1.873 | 1.84E-01 | -1.639 | 3.91E-01 | -1.272 | 3.74E-01 | 0.021 | 7.46E-01 | 1.306 | 5.17E-01 |
| CCL18 | 2.48 | 1.19E-03 | -0.541 | 5.48E-01 | 4.167 | 3.65E-04 | 2.311 | 6.17E-03 | 0.079 | 4.17E-02 | 2.477 | 2.93E-02 |
| CTAG2 | 9.88 | 1.58E-03 | 0.775 | 7.75E-01 | 10.105 | 3.93E-02 | 2.706 | 2.65E-01 | 0.271 | 2.65E-02 | 0.259 | 9.40E-01 |
| CDK1 | 1.70 | 1.77E-03 | 0.432 | 4.95E-01 | 1.676 | 4.57E-02 | 0.759 | 2.14E-01 | 0.001 | 9.82E-01 | -0.809 | 3.15E-01 |
| HOXA13 | 1.67 | 1.92E-03 | 0.102 | 8.90E-01 | -0.494 | 5.90E-01 | -0.225 | 7.39E-01 | 0.069 | 2.36E-02 | 1.566 | 8.43E-02 |
| CXCR2 | 1.22 | 2.28E-03 | -1.972 | 1.56E-03 | 2.973 | 4.16E-04 | 1.151 | 7.71E-02 | -0.022 | 4.56E-01 | -1.091 | 1.98E-01 |
| CTSE | 1.74 | 5.99E-03 | 1.226 | 1.77E-01 | 0.817 | 5.02E-01 | 0.095 | 9.13E-01 | 0.056 | 1.49E-01 | 3.004 | 7.93E-03 |
| SEMA3D | 3.07 | 8.70E-03 | -0.080 | 9.60E-01 | 5.333 | 2.77E-02 | 1.174 | 4.14E-01 | 0.171 | 1.17E-02 | -0.060 | 9.76E-01 |
| KLF9 | 1.17 | 8.97E-03 | -0.526 | 2.58E-01 | 2.297 | 2.19E-04 | 0.249 | 6.04E-01 | -0.016 | 4.74E-01 | -0.297 | 6.39E-01 |
| VEGFA | 0.49 | 1.17E-02 | -0.676 | 4.39E-02 | 0.422 | 3.37E-01 | -0.071 | 8.30E-01 | -0.005 | 7.21E-01 | -0.280 | 5.19E-01 |
| TERT | 3.46 | 1.74E-02 | 1.520 | 2.81E-01 | 6.206 | 3.85E-02 | 1.771 | 1.75E-01 | 0.101 | 1.36E-01 | -0.508 | 7.80E-01 |
| MMP10 | 1.49 | 3.66E-02 | 0.015 | 9.89E-01 | 4.042 | 3.06E-03 | 1.706 | 8.79E-02 | 0.046 | 3.29E-01 | -0.056 | 9.68E-01 |
| IGFBP5 | 1.13 | 4.61E-02 | 0.793 | 3.61E-01 | -0.127 | 9.07E-01 | -0.005 | 9.95E-01 | 0.063 | 8.70E-02 | 1.264 | 2.44E-01 |
| CCNE2 | 0.75 | 6.10E-02 | -0.259 | 5.63E-01 | 1.747 | 1.96E-03 | 0.828 | 4.29E-02 | -0.009 | 6.30E-01 | -0.131 | 8.15E-01 |
| ANG | -7.81 | 7.04E-02 | -16.899 | 9.98E-01 | 17.346 | 9.98E-01 | 17.458 | 9.98E-01 | -0.146 | 1.30E-01 | -4.224 | 6.42E-02 |
| SYNGR1 | 0.79 | 1.04E-01 | -0.481 | 4.44E-01 | -1.226 | 1.05E-01 | -0.503 | 3.72E-01 | 0.019 | 4.83E-01 | -0.441 | 5.71E-01 |
| CXCL1 | 0.49 | 1.42E-01 | -2.017 | 3.33E-04 | 1.562 | 4.08E-02 | 0.868 | 1.30E-01 | -0.031 | 2.23E-01 | -0.909 | 2.24E-01 |
| AHNAK2 | -0.61 | 2.26E-01 | 0.333 | 6.11E-01 | -0.612 | 4.72E-01 | -0.405 | 5.28E-01 | 0.028 | 3.26E-01 | -0.774 | 3.49E-01 |
| IL8 | 0.54 | 2.32E-01 | -2.289 | 2.75E-03 | 2.245 | 2.76E-02 | 0.607 | 4.39E-01 | -0.028 | 4.31E-01 | -0.708 | 4.87E-01 |
| APOE | 0.40 | 2.52E-01 | -0.366 | 4.52E-01 | 2.380 | 8.93E-05 | -0.069 | 8.88E-01 | 0.035 | 1.01E-01 | 0.484 | 4.45E-01 |
| AG | -0.59 | 4.33E-01 | -0.479 | 6.04E-01 | 3.844 | 1.12E-02 | 0.243 | 7.74E-01 | 0.055 | 1.74E-01 | 2.028 | 8.92E-02 |
| PRAME | 0.81 | 5.56E-01 | 1.540 | 4.08E-01 | 10.480 | 7.75E-03 | 2.355 | 2.20E-01 | 0.296 | 1.01E-03 | 0.711 | 7.76E-01 |
| PLAU | -0.06 | 8.72E-01 | -1.649 | 5.09E-03 | 1.829 | 1.82E-02 | 0.399 | 5.02E-01 | -0.028 | 2.96E-01 | -0.900 | 2.41E-01 |
| MXRA8 | 0.00 | 9.94E-01 | 0.830 | 1.89E-01 | -0.693 | 4.13E-01 | 0.362 | 5.32E-01 | 0.000 | 9.88E-01 | 0.685 | 4.02E-01 |
